# Discontinuation of Oral Anticoagulants in Atrial Fibrillation Patients: Impact of Treatment Strategy and on Patients’ Health Status

**DOI:** 10.3390/jcm12247712

**Published:** 2023-12-15

**Authors:** Ryo Nakamaru, Nobuhiro Ikemura, Takehiro Kimura, Yoshinori Katsumata, Charles F. Sherrod, Hiroshi Miyama, Yasuyuki Shiraishi, Hideaki Kanki, Koji Negishi, Ikuko Ueda, Keiichi Fukuda, Seiji Takatsuki, Shun Kohsaka

**Affiliations:** 1 Department of Cardiology, Keio University School of Medicine, Tokyo 160-8582, Japanwhite_cascade_libra@yahoo.co.jp (Y.S.);; 2 Department of Healthcare Quality Assessment, The University of Tokyo, Tokyo 113-8655, Japan; 3 Cardiovascular Research, Department of Biomedical and Health Informatics, Saint Luke’s Mid America Heart Institute/UMKC, Kansas City, MO 64111, USA; 4 Department of Cardiology, Saitama City Hospital, Saitama 336-8522, Japan; 5 Department of Cardiology, Yokohama Municipal Citizen’s Hospital, Yokohama 221-0855, Japan

**Keywords:** anticoagulant, direct-acting oral, atrial fibrillation, discontinuation, effectiveness, treatment, quality of life, warfarin

## Abstract

Aims: The discontinuation of oral anticoagulants (OACs) remains as a significant concern in the management of atrial fibrillation (AF). The discontinuation rate may vary depending on management strategy, and physicians may also discontinue OACs due to concerns about patient satisfaction with their care. We aimed to assess the incidence of OAC discontinuation and its relationship to patients’ health in an outpatient AF registry. Methods and Results: From a multicenter registry for newly recognized AF patients (*n* = 3313), we extracted 1647 (49.7%) patients with OACs and a CHA_2_DS_2_-Vasc score of ≥2. Discontinuation was defined as sustained cessation of OACs within a 1-year follow-up. We examined predictors associated with discontinuation and its relations to health status defined by the AFEQT questionnaire. Of the 1647 patients, 385 (23.6%) discontinued OACs after 1 year, with discontinuation rates varying across treatment strategies (15.3% for catheter ablation, 4.9% for rhythm control with antiarrhythmic drugs, and 3.0% for rate control). Successful rhythm control was associated with discontinuation in the catheter ablation (OR 6.61, 95% CI 3.00–14.6, *p* < 0.001) and antiarrhythmic drugs (OR 6.47, 95% CI 2.62–15.9, *p* < 0.001) groups, whereas the incidence of bleeding events within 1 year was associated with discontinuation in the rate control group. One-year AFEQT scores did not significantly differ between patients who discontinued OACs and those who did not in each treatment strategy group. Conclusions: OAC discontinuation was common among AF patients with significant stroke risk but varied depending on the chosen treatment strategy. This study also found no significant association between OAC discontinuation and patients’ health status.

## 1. Introduction

Current clinical guidelines recommend oral anticoagulants (OACs) for stroke prevention in patients with atrial fibrillation (AF), with an emphasis on adherence to OACs in follow-up [1]. Nonetheless, the annual incidence of OAC discontinuation is 10–15% in clinical trials [2,3] and is even higher in real-world settings [4,5,6,7,8,9,10]. Importantly, discontinuation leads to adverse events, and previous studies have demonstrated a 1.5–2.0-fold higher risk of subsequent stroke in patients who discontinued OACs [9,10,11,12].

Previous reports have revealed substantial OAC discontinuation rates in certain patient groups, such as those with older age, frailty, a lower risk of stroke, or a history of bleeding [4,7,8,9,10,13]. However, the reasons underlying the discontinuation of OACs are multifactorial [4,5] and may involve not only clinical factors, such as comorbidities, but also differences in treatment strategy and patient preferences that remain poorly characterized. In the current era, treatment decisions and patient outcomes can vary considerably depending on the chosen strategy (i.e., rhythm or rate control). Additionally, physicians may discontinue OACs due to concerns about patient satisfaction with their care during follow-up [14].

To address this gap in knowledge, we assessed the incidence of oral anticoagulant (OAC) discontinuation within one year of atrial fibrillation (AF) diagnosis, along with clinical factors related to discontinuation. Additionally, we conducted a comparison of health status using the Atrial Fibrillation Effect on Quality-of-Life (AFEQT) questionnaire between patients who discontinued OAC and those who did not in order to better understand the association between treatment strategy and discontinuation. Our findings may help optimize medical care and improve patients’ health status.

## 2. Methods

### 2.1. Data Sources

Data from the Keio Interhospital Cardiovascular Studies atrial fibrillation (KiCS-AF) registry were used. Details of the registry have been previously reported [15,16]. KiCS-AF recruited patients diagnosed with AF, either newly diagnosed or referred to outpatient clinics, from September 2012 to June 2018 at 11 institutions within the Tokyo metropolitan area of Japan. To capture patients’ health status before treatment initiation and response, we limited our participants to those who had been diagnosed with AF within the previous six months. Clinical research coordinators collected data on variables such as demographic information, anthropometric measurements, patient history, medication history, electrocardiography, and echocardiography records, as well as blood sampling test results for each patient, according to pre-defined criteria. The procedures followed were in accordance with institutional guidelines.

The number of registered cases (*n* = 3313) in this registry were determined based on the following considerations: To compare the HRQoL scores of patients reported in the four analysis groups (two study groups: direct oral anticoagulant [DOAC] and warfarin, and two strata: incident and prevalent), it was deemed necessary to have 1500 patients in each group with a 2-point difference in the mean scores between the two groups. This calculation assumes a standard deviation of 1, a two-sided α of 0.05, and an 80% power of detection [15].

Annual follow-up was conducted for all patients through chart reviews, mail, and phone interviews. As of now, we have successfully gathered 2-year follow-up data. Study coordinators updated information on major cardiovascular events and procedures, documented changes in laboratory test results and medications, and recorded responses from the AFEQT questionnaires. Data quality assurance was maintained through systematic validation, identifying outliers, and ensuring data completeness. Clinical research coordinators at each institution promptly addressed inquiries related to data entry. The senior study coordinator (I. U.) and investigator (S. K.) conducted on-site audits to verify accurate patient registration. This registry was approved by the Keio University School of Medicine Ethics Committee (reference number: 20120029) and the committee of each participating institution, and the research was conducted according to the Declaration of Helsinki. All participants provided written informed consent. Before the launch of this registry, information regarding the objectives of the study was provided for clinical trial registration with the University Hospital Medical Information Network (UMIN000022229).

### 2.2. Study Population and Discontinuation of OAC

**Figure 1:**
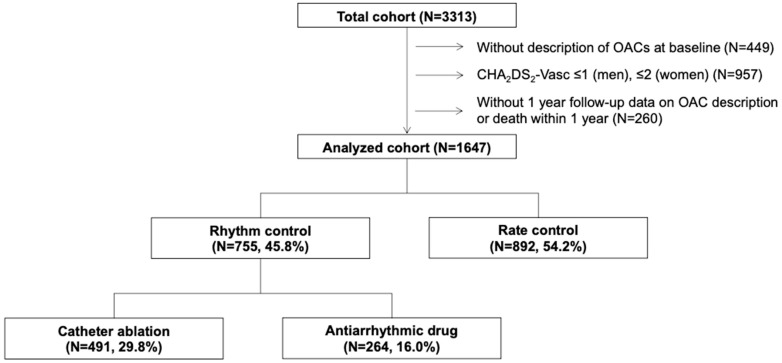
Study flowchart. OAC, oral anticoagulant.

From 3313 consecutive patients with AF in this registry, we extracted the data of 1907 (57.6%) patients who had an indication for OAC therapy (as determined by a CHA_2_DS_2_-Vasc score of ≥2 [≥3 for women] [1]) and received OACs (either DOAC or warfarin) at the time of registration. Among these patients, 1647 (86.4%) had available data for OAC status at the 1-year follow-up and were divided into three groups according to the chosen treatment strategy within 1 year after registration: (1) rhythm control with AF ablation (ablation group: *n* = 491, 29.5%); (2) rhythm control with antiarrhythmic drugs (AADs; AAD group: *n* = 254, 15.6%); and (3) those receiving rate control therapy, comprising those who had never received rhythm control (rate control group: *n* = 892, 54.2%; Figure 1). In this study, we defined the discontinuation of OAC therapy as sustained cessation of OAC therapy, as reported in the case report form at the 1-year follow-up.

### 2.3. Health Status Outcomes

All patients were requested to complete the AFEQT questionnaires at baseline and 1 year after enrollment in person or by mail. The AFEQT is a 20-item questionnaire that quantifies four domains of AF-related quality of life, including symptoms, daily activities, treatment concerns, and treatment satisfaction, using 7-point Likert scales [17]. The Atrial Fibrillation Effect on Quality-of-Life overall summary (AFEQT-OS) score is derived from the initial three domains and spans a scale from 0 to 100. A score of 0 indicates the most pronounced symptoms, physical limitations, and treatment concerns, while a score of 100 signifies the optimal atrial fibrillation-specific health status. For this study, we utilized a culturally and linguistically translated version of the AFEQT tailored for Japan.

### 2.4. Statistical Analysis

We compared baseline characteristics between patients who discontinued and continued OACs. Differences in normally distributed variables were evaluated using the Student’s *t*-test, and the Mann–Whitney U test was employed for non-normally distributed variables and their respective differences. Differences between independent categorical variables were assessed using the chi-square test. The trend of incidence of OAC discontinuation according to the CHA_2_DS_2_-Vasc score was assessed using the Cochrane–Armitage trend test.

To identify the contributing factors associated with OAC discontinuation for each group, we developed a generalized linear mixed model with a logit function, following adjustment for covariates including age, sex, CHA_2_DS_2_-Vasc score (men = 2 or women = 3 vs. men ≥ 3 or women ≥ 4), a modified HAS-BLED score (<3 vs. ≥3), which is the HAS-BLED score without labile international normalized ratio [18], DOAC or warfarin use, history of bleeding, bleeding events within 1 year after registration, and maintenance of sinus rhythm at 1-year follow-up (except for the rate control group).

We developed a general linear mixed model to compare the adjusted change in AFEQT scores, calculated as the score at the 1-year follow-up minus the score at registration, between patients who discontinued OAC and those who continued it. A positive change in AFEQT score signifies an improvement in HRQoL, whereas a negative change indicates a decline in HRQoL. The following covariates were selected based on clinical significance [19]: age, sex, body mass index, CHA_2_DS_2_-Vasc score (men = 2 or women = 3 vs. men ≥ 3 or women ≥ 4), DOAC or warfarin use, eGFR (>60 vs. ≤ 60 mL/min/1.73 m^2^), AFEQT baseline score, bleeding events within 1 year, and maintenance of sinus rhythm at 1-year follow-up (for the ablation and AAD groups) or type of AF (for the rate control group). We constructed scatter plots to examine a linearity assumption between the changes in AFEQT scores and continuous covariates. In the above two analyses, participating hospitals were included as random effects to account for the clustering of patients by site in the models. To account for missing values, we used single mean imputation (BMI; *n* = 14 [0.9%]) and excluded patients without data on the AFEQT-OS score at baseline (*n* = 4 [0.2%]) from the analyses.

Furthermore, we performed sensitivity analyses to assess the incidence of OAC discontinuation and to compare the changes in AFEQT-OS scores via excluding patients who experienced any bleeding events within 1 year (*n* = 38 [2.3%]), given that we were unable to eliminate bleeding events as potential confounding factors in the discontinuation of OAC and health status outcomes.

Finally, we compared the crude incidence of clinical adverse events, including all-cause death, stroke or transit ischemic attack, or any bleeding, within 2 years of follow-up between patients with OAC discontinuation and continuation. Since we defined OAC discontinuation as the absence of a prescription 1 year after registration, the first occurrence of an event (except for all-cause death) could precede OAC discontinuation. We employed a Cox proportional hazards model to explore the independent association between the discontinuation of OAC and the incidence of clinical adverse events, adjusting for age and CHA_2_DS_2_-Vasc score.

All reported *p*-values were two-sided, with *p* < 0.05 considered statistically significant. Standard error was used as the measure variance. All statistical analyses were performed using R software (version 4.2.2; R Project for Statistical Computing, Vienna, Austria).

## 3. Results

### 3.1. Incidence of OAC Discontinuation

**Figure 2:**
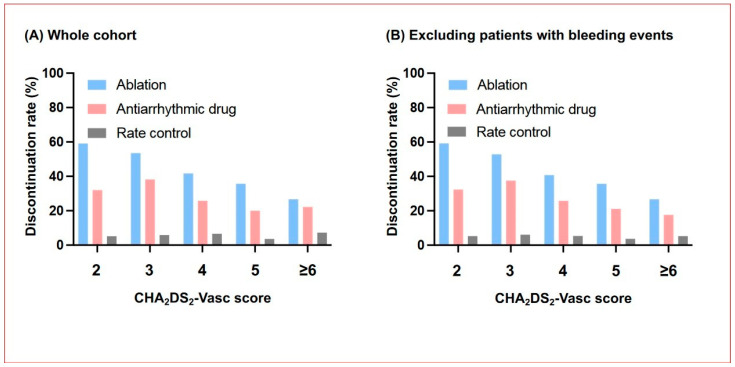
Incidence of OAC discontinuation within 1 year according to CHA_2_DS_2_-Vasc score. (**A**) Whole cohort (*n* = 1647). (**B**) Patients without bleeding events within 1 year (*n* = 1609). OAC, oral anticoagulant.

The baseline characteristics of the analytic cohort (*n* = 1647) are described in Table 1. A total of 385 (23.6%) patients discontinued OAC therapy within 1 year of registration. The discontinuation rates in the ablation, AAD, and rate control groups were 44.2%, 31.1%, and 5.7%, respectively (Figure 2A). The rates were not different across the CHA_2_DS_2_-Vasc scores, except in the ablation group (*p* for trend < 0.001). Among patients without bleeding events within 1 year, the rates in the ablation, AAD, and rate control groups were 51.0%, 30.8%, and 5.3%, respectively (Figure 2B). The rates also increased with lowering CHA_2_DS_2_-Vasc scores only in the ablation group (*p* for trend < 0.001).

The discontinuation rate was significantly higher in patients receiving DOAC therapy than those receiving warfarin therapy (24.7% vs. 17.0%, *p* < 0.006). While there was a higher discontinuation rate among patients receiving DOAC in the ablation group (DOAC 55.8%, warfarin 27.8%, *p* < 0.001), in both the AAD and rate control groups, there were no significant differences in discontinuation rates—AAD group: DOAC 31.3%, warfarin 29.8%, *p* = 0.97; rate control group: DOAC 5.2%, warfarin 8.8%, *p* = 0.23.

Patients who discontinued OACs were younger and had a lower mean CHA_2_DS_2_-Vasc score, were more paroxysmal AF, were more likely to be women, and were more likely to receive rhythm control compared with those who continued OACs (Table 1). In the ablation group, patients who discontinued OAC had a lower CHA_2_DS_2_-Vasc score compared to those who continued treatment (Table S1). In the AAD group, the patients who discontinued treatment were younger and had a lower mean CHA_2_DS_2_-Vasc score (Table S2). In contrast, the characteristics were similar between those with and without OAC continuation in the rate control groups (Table S3).

### 3.2. Clinical Factors Related to OAC Discontinuation

The maintenance of sinus rhythm at the 1-year follow-up was the strongest predictor of OAC discontinuation in both the ablation (OR 6.64, 95% CI 3.01–14.6, *p* < 0.001) and AAD (OR 6.34, 95% CI 2.61–15.4, *p* < 0.001) groups (Table 2 and Table 3, respectively). In addition, a lower CHA_2_DS_2_-Vasc score was a significant predictor in the ablation group, and being younger and female were significant predictors in the AAD group. The occurrence of bleeding within 1 year was significantly associated with discontinuation only in the rate control group (OR 4.47, 95% CI 1.58–12.6, *p* = 0.005, Table 4) but not in the ablation and AAD groups. A higher modified HAS-BLED score and a history of bleeding were not predictors in any group.

### 3.3. Health Status Outcomes and OAC Discontinuation

There was no significant difference in the baseline AFEQT-OS scores between patients who discontinued OAC and those who continued it across the three groups (Tables S1–S3). At the 1-year follow-up, among patients in the ablation and AAD groups, those who discontinued OAC within the first year were more likely to exhibit higher AFEQT-OS scores and subscale scores for daily activities, treatment satisfaction, and treatment concerns compared to those who continued OAC (Tables S1 and S2). In contrast, in the rate control group, these scores were comparable between the two groups (Table S3).

**Figure 3:**
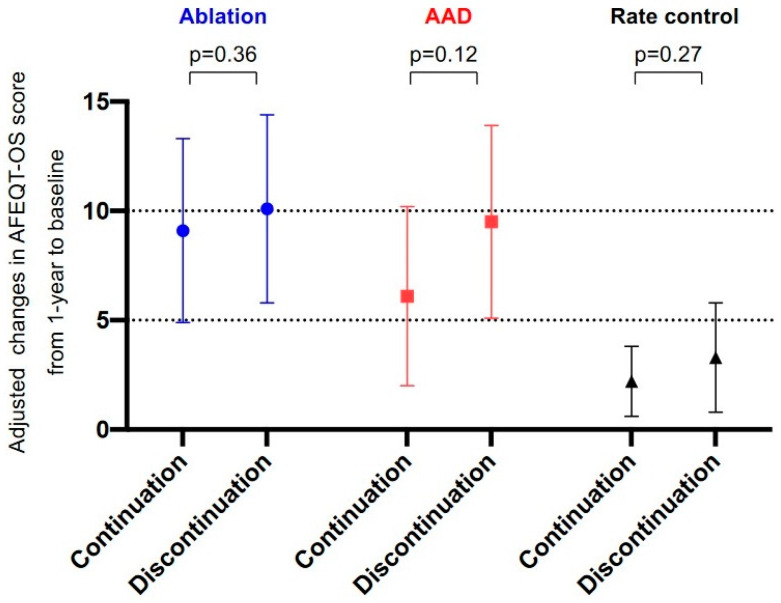
Comparison of adjusted AFEQT-OS score changes between OAC continuation and discontinuation from 1-year follow-up to baseline. Standard error is represented by the error bars. AAD, antiarrhythmic drug; AFEQT-OS, Atrial Fibrillation Effect on Quality-of-Life overall summary; OAC, oral anticoagulant.

The adjusted changes in AFEQT-OS score between 1 year and registration did not differ significantly between patients who discontinued OAC and those who continued it, regardless of the treatment strategy (mean [standard error], discontinuation vs. continuation: ablation, 10.1 [4.3] vs. 9.1 [4.2], *p* = 0.45; AAD, 9.5 [4.4] vs. 6.1 [4.1], *p* = 0.16; and rate control, 3.3 [2.5] vs. 2.2 [1.6], *p* = 0.27 (Figure 3)). The adjusted AFEQT subscales were also comparable between the groups, with the exception of the AAD group: (i) treatment satisfaction—ablation, 10.6 [4.2] vs. 6.7 [3.9], *p* = 0.07; AAD, 5.8 [5.6] vs. 0.2 [5.2], *p* = 0.07; and rate control, 4.4 [3.6] vs. 2.0 [2.3], *p* = 0.43; (ii) treatment concerns—ablation, 10.1 [3.0] vs. 9.7 [2.8], *p* = 0.75; AAD, 11.7 [4.7] vs. 6.8 [4.4], *p* = 0.06; and rate control, 4.8 [2.6] vs. 3.4 [1.6], *p* = 0.54).

Additionally, in patients without bleeding events within 1 year, the trend in comparison of the adjusted AFEQT-OS score was robust (discontinuation vs. continuation: ablation, 9.7 [3.7] vs. 8.7 [3.6], *p* = 0.44; AAD, 7.3 [2.8] vs. 4.1 [1.9], *p* = 0.19; and rate control, 4.3 [2.3] vs. 3.6 [0.8], *p* = 0.76).

### 3.4. Clinical Adverse Events

The crude incidence of death, stroke or transit ischemic attack, or bleeding during the 2-year follow-up is shown in Table S4. The incidence was similar between patients who continued OAC and those who discontinued OAC. The Cox proportional hazard model also suggested that OAC discontinuation was not independently associated with all-cause death (hazard ratio [HR] 1.03, 95% CI 0.29–3.67, *p* = 0.96), stroke (HR 0.95, 95% CI 0.26–3.39, *p* = 0.93), or bleeding (HR 0.90, 95% CI 0.44–1.84, *p* = 0.78) (Table S5).

## 4. Discussion

To gain a better understanding of the underlying causes of OAC discontinuation, we analyzed the incidence of OAC discontinuation within one year and identified clinical factors related to discontinuation among patients with newly diagnosed AF based on their initial treatment strategies. Approximately one-quarter of patients discontinued OAC therapy within 1 year, and the incidence was higher in the rhythm control group than in the rate control group. Of those who underwent ablation, maintenance of sinus rhythm and a low CHA_2_DS_2_-Vasc risk predicted discontinuation. Of those who received rate control therapy, bleeding risk predicted discontinuation. Finally, OAC discontinuation had little influence on health status outcome regardless of initial treatment strategy.

Given the substantial variation in the incidence of OAC discontinuation and its predictors based on the chosen initial treatment strategies, the present study offers novel insights into the underlying cause of OAC discontinuation. Particularly, we observed no difference in the discontinuation rates between patients treated with DOAC and those treated with warfarin, except in the ablation group. This finding contrasts with previous studies suggesting higher discontinuation rates in the DOAC group [5]. Considering the results regarding predictors of OAC discontinuation within each treatment group in this study, it may imply that physicians are more likely to discontinue OAC based on patient characteristics (i.e., embolic risk), treatment course, or bleeding events rather than the type of OAC when contemplating OAC discontinuation in patients undergoing pharmacotherapy (i.e., AAD or rate control therapy).

It is noteworthy that the discontinuation rate increased with a lower stroke risk among patients receiving ablation. These findings suggest that a substantial proportion of patients discontinued OACs based on the belief—whether held by the physicians or the patients or both—that reducing the AF burden would decrease stroke risk. Despite the fact that several studies have suggested a positive correlation between AF burden and stroke risk [20], there has remained an ongoing debate about OAC discontinuation following successful catheter ablation over the last decade. Nevertheless, existing data on this issue have been limited to observational studies and remained controversial [21,22,23,24,25,26]. In this present study, while we found no significant association between OAC discontinuation and clinical adverse events, we could not exclude that the finding was likely underpowered due to the small number of events. Thus, further investigations through long-term follow up data are warranted. Furthermore, a prior observational study emphasized that in patients with a higher CHA_2_DS_2_-VASc score, the possibility of not only embolic events but also left atrial appendage thrombus should be considered, even when under OAC therapy [27]. In these contexts, the current clinical guidelines emphasize that OAC treatment should be continued regardless of the maintenance of sinus rhythm [1]. Our findings highlight a substantial evidence–practice gap in the management of OAC treatment, particularly among patients undergoing catheter ablation.

To date, the details of the applied treatment strategy at the time of AF diagnosis and subsequent patient perceptions have been inadequately explored. The present study adds to prior studies by providing data on the influence of OAC discontinuation on health status outcomes in patients with AF. The treatment burden of patients with AF is increasing and is associated with poor health status outcomes [1]. Therapeutic burdens impair adherence, and this may be present in up to a quarter of patients [1,28]. While these burdens may manifest as the number and frequency of procedures and rate control titration, the choice of anticoagulant adds to the burden as well. While a previous study has shown that patients who are anticoagulated with warfarin are more likely to face a greater treatment burden than those who take DOAC [5], we found that health status outcomes between patients who discontinued and those who continued OAC were similar across each chosen treatment strategy. Approximately 80% of patients were prescribed DOAC and may have contributed to this finding. The present study indicates that the discontinuation of OAC therapy may have a low impact on health status outcomes even in patients without bleeding events, emphasizing that anticoagulation management should be determined based on individual clinical characteristics. Moreover, we provide crucial information for both physicians and patients in decision-making processes regarding the appropriate utilization of OACs.

This study has several limitations. First, since this was an observational study, the treatment strategy (e.g., rhythm or rate control) was based on physician judgment, which may have introduced selection bias and influenced the outcomes in the study population. Second, we did not have detailed data regarding the duration of OAC discontinuation. While OAC discontinuation was defined as continuous cessation of OAC therapy based on the case report form at the 1-year follow-up, we could not assess the influence of OAC discontinuation duration on clinical and health status outcomes. Third, we could not assess frailty, which is a significant factor of OAC discontinuation [13]. This limitation would affect the findings of the factors associated with the discontinuation. Fourth, because we were unable to identify the date of OAC discontinuation, the first occurrence of an event other than all-cause death may have preceded OAC discontinuation, which may have led to an overestimation of adverse clinical events due to OAC persistence. Finally, since the analyzed patients were recruited via the KiCS-AF registry, there is a potential for sampling bias, and the generalizability of the findings in this study raises valid concerns.

## 5. Conclusions

Approximately a quarter of patients with AF who had an indication for OAC therapy discontinued OACs within 1 year, with a higher incidence among those undergoing catheter ablation. Given that we found no significant differences in health status outcomes between patients who discontinued OAC and those who continued it, the clinical decision on OAC therapy should be based on individual stroke risk. Persistent efforts are required for the appropriate utilization of OACs.

## Figures and Tables

**Table 1:** Patient characteristics.

	All Patients (*n* = 1647)	Continuation (*n* = 1262)	Discontinuation (*n* = 385)	*p*-Value
Age, years	73.3 (8.3)	74.0 (8.2)	71.1 (8.0)	<0.001
Female	584 (35.5%)	455 (36.1%)	129 (33.5%)	0.39
Body mass index, kg/m^2^	23.6 (3.7)	23.6 (3.8)	23.6 (3.7)	0.99
Hemoglobin, g/dL	13.6 (1.8)	13.6 (1.8)	13.7 (1.7)	0.24
eGFR, mL/min/1.73 m^2^	58.6 (16.4)	58.1 (16.5)	60.1 (16.0)	0.039
Left ventricular ejection fraction, %	62.0 (12.4)	61.4 (13.2)	63.6 (9.1)	0.004
Left atrium diameter, cm	4.3 (0.8)	4.4 (0.8)	4.0 (0.7)	<0.001
**Type of atrial fibrillation**				
First detected	86 (5.2%)	72 (5.7%)	14 (3.6%)	<0.001
Paroxysmal	730 (44.3%)	493 (39.3%)	237 (61.6%)	
Persistent	454 (27.6%)	355 (28.3%)	99 (25.7%)	
Permanent	342 (20.8%)	309 (24.6%)	33 (8.6%)	
Unknown	35 (2.1%)	26 (2.1%)	2 (0.5%)	
**Comorbidities**				
Heart failure	455 (27.6%)	393 (31.3%)	62 (16.1%)	<0.001
Hypertension	1264 (76.8%)	955 (75.7%)	309 (80.3%)	0.07
Diabetes	418 (25.4%)	335 (26.5%)	83 (21.6%)	0.06
Previous Stroke	231 (14.0%)	191 (15.1%)	44 (11.4%)	0.024
Coronary artery disease	210 (12.8%)	179 (14.2%)	31 (8.1%)	0.002
Previous cerebral bleeding	29 (1.8%)	25 (2.0%)	4 (1.0%)	0.31
Previous GI bleeding	25 (1.5%)	20 (1.6%)	5 (1.3%)	0.87
**CHA_2_DS_2_-Vasc score**				
Mean	3.5 (1.3)	3.6 (1.3)	3.1 (1.2)	<0.001
7–9	46 (2.8%)	38 (3.0%)	8 (2.1%)	<0.001
4–6	663 (40.3%)	563 (44.6%)	100 (26.0%)	
3	504 (30.6%)	370 (29.3%)	134 (34.8%)	
2	434 (26.4%)	291 (23.1%)	143 (37.1%)	
mHAS_BLED score	2 (1–2)	2 (1–2)	2 (1–2)	0.08
**Treatment strategy**				
Rate control	892 (54.2%)	841 (66.6%)	51 (13.2%)	<0.001
Rhythm control	755 (45.8%)	421 (33.4%)	334 (86.8%)	
Ablation within 1-year	491 (29.8%)	239 (18.9%)	252 (65.5%)	<0.001
**Medications**				
DOAC	1359 (82.5%)	1023 (81.1%)	336 (87.3%)	0.006
Warfarin	288 (17.5%)	239 (18.9%)	49 (12.7%)	
Antiplatelet therapy	291 (17.7%)	237 (18.8%)	54 (14.0%)	0.06
β-blocker	975 (59.2%)	764 (60.5%)	211 (54.8%)	0.052
ACE-I/ARB	818 (49.7%)	644 (51.1%)	174 (45.2%)	0.052
Ca-blocker	834 (50.6%)	632 (50.1%)	202 (52.5%)	0.45
Digoxin	122 (7.4%)	111 (8.8%)	11 (2.9%)	<0.001
Pilsicainide	91 (5.5%)	61 (4.8%)	30 (7.8%)	0.036
Bepridil	73 (4.4%)	47 (3.7%)	26 (6.8%)	0.017
Flecainide	39 (2.4%)	28 (2.2%)	11 (2.9%)	0.60
**AFEQT**				
**Baseline**				
Overall summary	76.9 (17.8)	77.3 (17.5)	75.5 (18.5)	0.08
Symptom	80.6 (19.4)	81.7 (18.8)	77.2 (20.9)	<0.001
Daily activities	74.3 (22.5)	74.1 (22.5)	74.8 (22.4)	0.57
Treatment concerns	77.7 (18.5)	78.5 (18.1)	75.2 (19.3)	0.003
Treatment satisfaction	69.5 (19.4)	69.6 (19.3)	69.2 (20.0)	0.76
**1-year**				
Overall summary	83.8 (15.0)	82.5 (15.1)	88.0 (13.8)	<0.001
Symptom	87.9 (14.7)	87.2 (14.6)	90.0 (15.1)	0.002
Daily activities	80.4 (20.0)	78.7 (20.3)	86.1 (17.6)	<0.001
Treatment concerns	85.4 (14.8)	84.3 (14.9)	88.9 (13.7)	<0.001
Treatment satisfaction	78.0 (18.4)	75.5 (18.3)	86.3 (16.3)	<0.001

Values are median (IQR), or *n* (%). The P-values indicate differences between patients with continuation and those with discontinuation. ACE-I, angiotensin converting enzyme inhibitor; AFEQT, Atrial Fibrillation Effect on Quality of Life; ARB, angiotensin receptor blocker; DOAC, direct oral anticoagulant; eGFR, estimated glomerular filtration rate; GI, gastrointestinal.

**Table 2:** Factors associated with discontinuation of OACs in the ablation group.

Variables	Odds Ratio (95% CI)	*p*-Value
Age (per 1 year increase)	1.02 (0.99–1.06)	0.16
Female Sex	0.64 (0.39–1.04)	0.07
Lower CHA_2_DS_2_-Vasc score (men = 2 or women = 3)	2.92 (1.78–4.79)	<0.001
Higher modified HAS-BLED score (≥3)	0.96 (0.49–1.89)	0.91
DOAC (vs. warfarin)	2.53 (1.32–4.87)	0.005
Maintenance of sinus rhythm at 1 year	6.64 (3.01–14.6)	<0.001
History of bleeding	1.37 (0.32–5.92)	0.67
Occurrence of bleeding events within 1 year	1.92 (0.39–9.51)	0.42

AFEQT-OS, Atrial Fibrillation Effect on Quality-of-Life overall summary; CI, confidence interval; DOAC, direct oral anticoagulant.

**Table 3:** Factors associated with discontinuation of OACs in the antiarrhythmic drug group.

Variables	Odds Ratio (95% CI)	*p*-Value
Age (per 1 year increase)	0.93 (0.88–0.97)	0.002
Female Sex	2.97 (1.43–6.17)	0.003
Lower CHA_2_DS_2_-Vasc score (men = 2 or women = 3)	1.19 (0.56–2.52)	0.65
Higher modified HAS-BLED score (≥3)	0.80 (0.29–2.24)	0.67
DOAC (vs. warfarin)	1.59 (0.60–4.17)	0.35
Maintenance of sinus rhythm at 1 year	6.32 (2.59–15.4)	<0.001
History of bleeding	0.89 (0.15–5.41)	0.90
Occurrence of bleeding events within 1 year	3.69 (0.33–41.9)	0.29

AFEQT-OS, Atrial Fibrillation Effect on Quality-of-Life overall summary; CI, confidence interval; DOAC, direct oral anticoagulant.

**Table 4:** Factors associated with discontinuation of OACs in the rate control group.

Variables	Odds Ratio (95% CI)	*p*-Value
Age (per 1 year increase)	1.00 (0.96–1.05)	0.84
Female Sex	1.21 (0.67–2.19)	0.53
Lower CHA_2_DS_2_-Vasc score (men = 2 or women = 3)	0.91 (0.44–1.88)	0.80
Higher modified HAS-BLED score (≥3)	0.95 (0.45–2.01)	0.90
DOAC (vs. warfarin)	0.62 (0.32–1.19)	0.15
History of bleeding	0.94 (0.21–4.23)	0.94
Occurrence of bleeding events within 1 year	4.47 (1.59–12.6)	0.004

AFEQT-OS, Atrial Fibrillation Effect on Quality-of-Life overall summary; CI, confidence interval; DOAC, direct oral anticoagulant.

## Data Availability

The data underlying this article will be shared on reasonable request from the corresponding author.

## Supplementary Materials

The following supporting information can be downloaded at: https://www.mdpi.com/article/10.3390/jcm12247712/s1, Table S1: Comparison of patient characteristics between continuation and discontinuation of OAC in the ablation group. Table S2: Comparison of patient characteristics between continuation and discontinuation of OAC in the antiarrhythmic drugs group. Table S3: Comparison of patient characteristics between continuation and discontinuation of OAC in the rate control group. Table S4: Crude incidence of adverse clinical events during 2 years after registration. Table S5: Association OAC discontinuation and clinical adverse events during 2 years after registration.

## References

[1] Hindricks G., Potpara T., Dagres N., Arbelo E., Bax J.J., Blomström-Lundqvist C., Boriani G., Castella M., Dan G.A., Dilaveris P.E. (2021). 2020 ESC Guidelines for the diagnosis and management of atrial fibrillation developed in collaboration with the European Association for Cardio-Thoracic Surgery (EACTS). Eur. Heart J..

[2] Connolly S.J., Ezekowitz M.D., Yusuf S., Eikelboom J., Oldgren J., Parekh A., Pogue J., Reilly P.A., Themeles E., Varrone J. (2009). Dabigatran versus Warfarin in Patients with Atrial Fibrillation. N. Engl. J. Med..

[3] Granger C.B., Alexander J.H., McMurray J.J., Lopes R.D., Hylek E.M., Hanna M., Al-Khalidi H.R., Ansell J., Atar D., Avezum A. (2011). Apixaban versus Warfarin in Patients with Atrial Fibrillation. N. Engl. J. Med..

[4] Buck J., Fromings Hill J., Martin A., Springate C., Ghosh B., Ashton R., Lee G., Orlowski A. (2021). Reasons for discontinuing oral anticoagulation therapy for atrial fibrillation: A systematic review. Age Ageing.

[5] O’Brien E.C., Simon D.N., Allen L.A., Singer D.E., Fonarow G.C., Kowey P.R., Thomas L.E., Ezekowitz M.D., Mahaffey K.W., Chang P. (2014). Reasons for warfarin discontinuation in the Outcomes Registry for Better Informed Treatment of Atrial Fibrillation (ORBIT-AF). Am. Heart J..

[6] Vedovati M.C., Verdecchia P., Giustozzi M., Molini G., Conti S., Pierpaoli L., Valecchi F., Aita A., Agnelli G., Becattini C. (2017). Permanent discontinuation of non vitamin K oral anticoagulants in real life patients with non-valvular atrial fibrillation. Int. J. Cardiol..

[7] Ferroni E., Gennaro N., Costa G., Fedeli U., Denas G., Pengo V., Corti M.C. (2019). Real-world persistence with direct oral anticoagulants (DOACs) in naïve patients with non-valvular atrial fibrillation. Int. J. Cardiol..

[8] Jackson L.R., Kim S., Blanco R., Thomas L., Ansell J., Fonarow G.C., Gersh B.J., Go A.S., Kowey P.R., Mahaffey K.W. (2020). Discontinuation rates of warfarin versus direct acting oral anticoagulants in US clinical practice: Results from Outcomes Registry for Better Informed Treatment of Atrial Fibrillation II (ORBIT-AF II). Am. Heart J..

[9] Cools F., Johnson D., Camm A.J., Bassand J.P., Verheugt F.W.A., Yang S., Tsiatis A., Fitzmaurice D.A., Goldhaber S.Z., Kayani G. (2021). Risks associated with discontinuation of oral anticoagulation in newly diagnosed patients with atrial fibrillation: Results from the GARFIELD-AF Registry. J. Thromb. Haemost..

[10] Toorop M.M.A., Chen Q., Tichelaar V.Y.I.G., Cannegieter S.C., Lijfering W.M. (2021). Predictors, time course, and outcomes of persistence patterns in oral anticoagulation for non-valvular atrial fibrillation: A Dutch Nationwide Cohort Study. Eur. Heart J..

[11] García Rodríguez L.A., Cea Soriano L., Munk Hald S., Hallas J., Balabanova Y., Brobert G., Vora P., Sharma M., Gaist D. (2021). Discontinuation of oral anticoagulation in atrial fibrillation and risk of ischaemic stroke. Heart.

[12] Ozaki A.F., Choi A.S., Le Q.T., Ko D.T., Han J.K., Park S.S., Jackevicius C.A. (2020). Real-World Adherence and Persistence to Direct Oral Anticoagulants in Patients With Atrial Fibrillation: A Systematic Review and Meta-Analysis. Circ. Cardiovasc. Qual. Outcomes.

[13] Romiti G.F., Proietti M., Bonini N., Ding W.Y., Boriani G., Huisman M.V., Lip G.Y.H., GLORIA-AF Investigators (2022). Clinical Complexity Domains, Anticoagulation, and Outcomes in Patients with Atrial Fibrillation: A Report from the GLORIA-AF Registry Phase II and III. Thromb. Haemost..

[14] Cannon C.P., Kim J.M., Lee J.J., Sutherland J., Bachireddy R., Valentine C.M., Hearne S., Trebnick A., Jaffer S., Datta S. (2023). Patients and Their Physician’s Perspectives About Oral Anticoagulation in Patients with Atrial Fibrillation Not Receiving an Anticoagulant. JAMA Netw. Open.

[15] Ikemura N., Spertus J.A., Kimura T., Mahaffey K., Piccini J.P., Inohara T., Ueda I., Tanimoto K., Suzuki M., Nakamura I. (2019). Cohort profile: Patient characteristics and quality-of-life measurements for newly-referred patients with atrial fibrillation—Keio interhospital Cardiovascular Studies-atrial fibrillation (KiCS-AF). BMJ Open.

[16] Nakamaru R., Ikemura N., Spertus J.A., Kimura T., Katsumata Y., Fujisawa T., Ueno K., Inoue S., Ueda I., Fukuda K. (2022). Rate versus rhythm control in patients with newly diagnosed atrial fibrillation: Effects of the treatment timing on health status outcomes. Am. Heart J..

[17] Spertus J., Dorian P., Bubien R., Lewis S., Godejohn D., Reynolds M.R., Lakkireddy D.R., Wimmer A.P., Bhandari A., Burk C. (2011). Development and Validation of the Atrial Fibrillation Effect on QualiTy-of-Life (AFEQT) Questionnaire in Patients With Atrial Fibrillation. Circ. Arrhythm. Electrophysiol..

[18] Yao X., Abraham N.S., Sangaralingham L.R., Bellolio M.F., McBane R.D., Shah N.D., Noseworthy P.A. (2016). Effectiveness and Safety of Dabigatran, Rivaroxaban, and Apixaban Versus Warfarin in Nonvalvular Atrial Fibrillation. J. Am. Heart Assoc..

[19] Ikemura N., Kohsaka S., Kimura T., Ueda I., Katsumata Y., Nishiyama T., Aizawa Y., Tanimoto K., Momiyama Y., Akaishi M. (2019). Assessment of Sex Differences in the Initial Symptom Burden, Applied Treatment Strategy, and Quality of Life in Japanese Patients with Atrial Fibrillation. JAMA Netw. Open.

[20] Tiver K.D., Quah J., Lahiri A., Ganesan A.N., McGavigan A.D. (2021). Atrial fibrillation burden: An update—The need for a CHA2DS2-VASc-AFBurden score. Europace.

[21] Chew D., Piccini J.P. (2021). Long-term oral anticoagulant after catheter ablation for atrial fibrillation. Europace.

[22] Oral H., Chugh A., Ozaydin M., Good E., Fortino J., Sankaran S., Reich S., Igic P., Elmouchi D., Tschopp D. (2006). Risk of Thromboembolic Events after Percutaneous Left Atrial Radiofrequency Ablation of Atrial Fibrillation. Circulation.

[23] Själander S., Holmqvist F., Smith J.G., Platonov P.G., Kesek M., Svensson P.J., Blomström-Lundqvist C., Tabrizi F., Tapanainen J., Poci D. (2017). Assessment of Use vs Discontinuation of Oral Anticoagulation after Pulmonary Vein Isolation in Patients With Atrial Fibrillation. JAMA Cardiol..

[24] Kadire S.R., Al-Khatib S.M., Calkins H. (2021). Anticoagulation after Ablation for Atrial Fibrillation. N. Engl. J. Med..

[25] Karasoy D., Gislason G.H., Hansen J., Johannessen A., Køber L., Hvidtfeldt M., Özcan C., Torp-Pedersen C., Hansen M.L. (2015). Oral anticoagulation therapy after radiofrequency ablation of atrial fibrillation and the risk of thromboembolism and serious bleeding: Long-term follow-up in nationwide cohort of Denmark. Eur. Heart J..

[26] Liu X.H., Xu Q., Luo T., Zhang L., Liu H.J. (2021). Discontinuation of oral anticoagulation therapy after successful atrial fibrillation ablation: A systematic review and meta-analysis of prospective studies. PLoS ONE.

[27] Di Cori A., Barletta V., Meola L., Parollo M., Mazzocchetti L., Carluccio M., Branchitta G., Cellamaro T., Gentile F., Segreti L. (2022). Left atrial thrombus and smoke resolution in patients with atrial fibrillation under chronic oral anticoagulation. J. Interv. Card. Electrophysiol..

[28] Potpara T.S., Mihajlovic M., Zec N., Marinkovic M., Kovacevic V., Simic J., Kocijancic A., Vajagic L., Jotic A., Mujovic N. (2020). Self-reported treatment burden in patients with atrial fibrillation: Quantification, major determinants, and implications for integrated holistic management of the arrhythmia. Europace.

