# Supplementary material for: Discontinuation of Oral Anticoagulants in Atrial Fibrillation Patients: Impact of Treatment Strategy and on Patients’ Health Status
Source: J Clin Med. 2023 Dec 15;12(24):7712. doi: 10.3390/jcm12247712 (PMC10743485; doi:10.3390/jcm12247712)
Supplement: Supplementary file 1 [file jcm-12-07712-s001.zip › jcm-2738941-supplementary.pdf]

Supplemental Material

Discontinuation of Oral Anticoagulants in Atrial Fibrillation Patients: Impact of Treatment Strategy and on Patients' Health Status

Table S1. Comparison of patient characteristics between continuation and discontinuation of OAC in the ablation group

Table S2. Comparison of patient characteristics between continuation and discontinuation of OAC in the antiarrhythmic drugs group

Table S3. Comparison of patient characteristics between continuation and discontinuation of OAC in the rate control group

Table S4. Crude incidence of adverse clinical events during 2 years after registration

Table S5. Association OAC discontinuation and clinical adverse events during 2 years after registration

Table S1. Comparison of patient characteristics between continuation and discontinuation of OAC in the ablation group

	Continuation (n=239)	Discontinuation (n=252)	P-Value
Age, years	69.5 (7.5)	70.2 (7.9)	0.32
Female	75 (31.4%)	70 (27.8%)	0.44
Body mass index, kg/m^2	24.0 (3.5)	23.8 (3.5)	0.35
Hemoglobin, g/dL	14.3 (1.6)	14.0 (1.6)	0.13
eGFR, mL/min/1.73m^2	60.1 (13.7)	60.5 (15.7)	0.80
**Type of AF**			
First detected	4 (1.7%)	5 (2.0%)	
Paroxysmal	116 (48.5%)	167 (66.3%)	
Persistent	82 (34.3%)	64 (25.4%)	<0.001
Permanent	35 (14.6%)	16 (6.3%)	
Unknown	2 (0.8%)	0	
**Comorbidities**			
Heart failure	42 (17.6%)	25 (9.9%)	0.019
Hypertension	196 (82.0%)	208 (82.5%)	0.97
Diabetes	58 (24.3%)	53 (21.0%)	0.45
Previous Stroke	55 (23.0%)	22 (8.7%)	<0.001
Coronary artery disease	24 (10.0%)	20 (7.9%)	0.51
Previous cerebral bleeding	6 (2.5%)	2 (0.8%)	0.25
Previous GI bleeding	1 (0.4%)	3 (1.2%)	0.65
**CHA_2DS_2-Vasc score**			
Mean score	3.2 (1.1)	2.9 (1.0)	<0.001
7-9	1 (0.4%)	1 (0.4%)	<0.001
4-6	88 (36.8%)	56 (22.2%)	
3	74 (31.0%)	85 (33.7%)	
2	76 (31.8%)	110 (43.7%)	

mHAS_BLED score	2 (1-2)	2 (1-2)	0.85
**Echocardiographic data**			
LVDD, cm	4.7 (0.6)	4.6 (0.6)	0.26
LVEF, %	62.4 (11.8)	63.7 (8.9)	0.18
Left atrium diameter, cm	4.2 (0.8)	3.9 (0.7)	<0.001
**Medications**			
DOAC	182 (76.2%)	230 (91.3%)	<0.001
Warfarin	57 (23.8%)	22 (8.7%)	
Antiplatelet therapy	30 (12.6%)	34 (13.5%)	0.86
β-blocker	139 (58.2%)	138 (54.8%)	0.50
ACE-I/ARB	124 (51.9%)	123 (48.8%)	0.56
Ca-blocker	132 (55.2%)	141 (56.0%)	0.94
Digoxin	13 (5.4%)	5 (2.0%)	0.07
**AFEQT-OS score**			
**Baseline**			
Overall summary	74.1 (18.5)	74.6 (18.9)	0.78
Symptoms	76.0 (20.2)	74.5 (21.9)	0.43
Daily activities	73.5 (22.7)	75.3 (22.7)	0.40
Treatment satisfactions	62.9 (21.2)	65.9 (20.3)	0.16
Treatment concerns	72.8 (19.4)	73.9 (19.3)	0.54
**1-year**			
Overall summary	86.9 (13.0)	89.2 (13.3)	0.06
Symptoms	89.2 (14.6)	90.1 (15.0)	0.50
Daily activities	85.8 (16.2)	88.1 (16.1)	0.12
Treatment satisfactions	81.0 (21.0)	88.3 (15.2)	<0.001
Treatment concerns	86.9 (13.7)	89.8 (13.2)	0.019

Values are mean (SD), median (IQR), or n (%). ACE-I, angiotensin converting enzyme inhibitor; AF, atrial fibrillation; AFEQT, Atrial Fibrillation Effect on Quality-of-Life; ARB, angiotensin receptor blocker; BNP, brain natriuretic peptide; DOAC, direct oral anticoagulant; eGFR, estimated glomerular filtration rate; GI, gastrointestinal; LVDD, left ventricular end-diastolic dimension; LVEF, left ventricular ejection fraction; mHAS-BLED; modified HAS-BLED.

Table S2. Comparison of patient characteristics between continuation and discontinuation of OAC in the antiarrhythmic drugs group

	Continuation (n=182)	Discontinuation (n=82)	P-Value
Age, years	74.2 (8.1)	70.7 (8.2)	0.002
Female	66 (36.3%)	20 (24.4%)	0.37
Body mass index, kg/m^2	24.0 (3.7)	23.9 (4.4)	0.31
Family history of AF	67 (15.9%)	58 (17.4%)	0.86
Hemoglobin, g/dL	13.4 (1.6)	13.2 (1.4)	0.24
eGFR, mL/min/1.73m^2	58.3 (17.4)	62.1 (14.6)	0.09
**Type of AF**			
First detected	10 (5.5%)	3 (3.7%)	
Paroxysmal	112 (61.5%)	54 (65.9%)	
Persistent	46 (25.3%)	23 (28.0%)	0.35
Permanent	14 (7.7%)	2 (2.4%)	
Unknown	0	0	
**Comorbidities**			
Heart failure	34 (18.7%)	20 (24.4%)	0.37
Hypertension	133 (73.1%)	61 (74.4%)	0.94
Diabetes	45 (24.7%)	20 (24.4%)	>0.99
Previous Stroke	25 (13.7%)	9 (11.0%)	0.67
Coronary artery disease	18 (9.9%)	8 (9.8%)	>0.99
Previous cerebral bleeding	4 (2.2%)	1 (1.2%)	0.96
Previous GI bleeding	5 (2.7%)	1 (1.2%)	0.75
**CHA_2DS_2-Vasc score**			
Mean score	3.3 (1.2)	3.0 (1.1)	<0.001
7-9	4 (2.2%)	3 (3.6%)	0.13
4-6	72 (39.6%)	21 (25.6%)	
3	55 (30.2%)	34 (41.5%)	

2	51 (28.0%)	24 (29.3%)	
mHAS_BLED score	2 (1-2)	2 (1-2)	0.35
**Echocardiographic data**			
LVDD, cm	4.6 (0.6)	4.6 (0.6)	0.91
LVEF, %	64.7 (10.2)	63.3 (8.8)	0.29
Left atrium diameter, cm	4.2 (0.7)	3.9 (0.6)	<0.001
**Medications**			
DOAC	149 (81.9%)	68 (82.9%)	0.97
Warfarin	33 (18.1%)	14 (17.1%)	
Antiplatelet therapy	31 (17.0%)	10 (12.2%)	0.41
β-blocker	101 (55.5%)	40 (48.8%)	0.38
ACE-I/ARB	88 (48.4%)	31 (37.8%)	0.14
Ca-blocker	96 (52.7%)	39 (47.6%)	0.52
Digoxin	7 (3.8%)	0	0.17
Pilsicainide	28 (15.4%)	6 (7.3%)	0.11
Flecainide	10 (5.5%)	3 (3.7%)	0.74
Cibenzoline	13 (7.1%)	2 (2.4%)	0.22
Bepridil	18 (9.9%)	10 (12.2%)	0.73
**AFEQT-OS score**			
**Baseline**			
Overall summary	74.1 (18.5)	76.6 (19.2)	0.32
Symptoms	78.5 (21.5)	81.4 (19.8)	0.31
Daily activities	70.6 (24.6)	75.1 (23.1)	0.16
Treatment satisfactions	67.9 (18.1)	76.6 (18.3)	0.001
Treatment concerns	75.7 (17.6)	75.5 (20.2)	0.95
**1-year**			
Overall summary	79.2 (16.8)	86.6 (12.9)	0.001
Symptoms	83.1 (17.6)	88.9 (16.8)	0.018
Daily activities	76.1 (21.6)	84.7 (16.7)	0.003
Treatment satisfactions	73.7 (18.3)	84.8 (16.9)	<0.001
Treatment concerns	80.8 (16.7)	87.4 (13.8)	0.003

Values are mean (SD), median (IQR), or n (%). ACE-I, angiotensin converting enzyme

inhibitor; AF, atrial fibrillation; AFEQT, Atrial Fibrillation Effect on Quality-of-Life; ARB, angiotensin receptor blocker; BNP, brain natriuretic peptide; DOAC, direct oral anticoagulant; eGFR, estimated glomerular filtration rate; GI, gastrointestinal; LVDD, left ventricular end-diastolic dimension; LVEF, left ventricular ejection fraction; mHAS-BLED; modified HAS-BLED; OAC oral anticoagulant.

Table S3. Comparison of patient characteristics between continuation and discontinuation of OAC in the rate control group

	Continuation (n=841)	Discontinuation (n=51)	P-Value
Age, years	75.2 (8.0)	75.8 (7.0)	0.57
Female	317 (37.7%)	17 (33.3%)	0.63
Body mass index, kg/m^2	23.5 (3.9)	22.3 (3.0)	0.027
Hemoglobin, g/dL	13.4 (1.8)	12.9 (2.0)	0.042
eGFR, mL/min/1.73m^2	57.5 (17.1)	54.9 (19.0)	0.31
**Type of AF**			
First detected	58 (7.0%)	6 (11.8%)	
Paroxysmal	265 (31.8%)	16 (31.4%)	
Persistent	227 (27.2%)	12 (23.5%)	0.73
Permanent	260 (31.2%)	15 (29.4%)	
Unknown	24 (2.9%)	2 (3.9%)	
**Comorbidities**			
Heart failure	317 (37.3%)	17 (33.3%)	0.63
Hypertension	626 (74.4%)	40 (78.4%)	0.64
Diabetes	232 (27.6%)	10 (19.6%)	0.28
Previous Stroke	111 (13.2%)	9 (17.6%)	0.49
Coronary artery disease	24 (10.0%)	20 (7.9%)	0.51
Previous cerebral bleeding	6 (2.5%)	2 (0.8%)	0.25
Previous GI bleeding	1 (0.4%)	3 (1.2%)	0.65
**CHA_2DS_2-Vasc score**			
Mean score	3.7 (1.4)	3.8 (1.4)	0.76
7-9	33 (3.9%)	4 (7.8%)	0.63
4-6	403 (47.9%)	23 (45.1%)	
3	241 (28.7%)	15 (29.4%)	
2	164 (19.5%)	9 (17.6%)	

mHAS_BLED score	2 (1-2)	2 (1-2)	0.94
**Echocardiographic data**			
LVDD, cm	4.8 (0.7)	4.6 (0.6)	0.13
LVEF, %	60.4 (14.1)	63.7 (10.4)	0.13
Left atrium diameter, cm	4.4 (0.8)	4.4 (0.8)	0.44
**Medications**			
DOAC	692 (82.3%)	38 (74.5%)	0.23
Warfarin	149 (17.7%)	13 (25.5%)	
Antiplatelet therapy	176 (20.9%)	10 (19.6%)	0.96
β-blocker	524 (62.3%)	33 (64.7%)	0.85
ACE-I/ARB	432 (51.4%)	20 (39.2%)	0.12
Ca-blocker	404 (48.0%)	22 (43.1%)	0.59
Digoxin	91 (10.8%)	6 (11.8%)	>0.99
**AFEQT-OS score**			
**Baseline**			
Overall summary	78.9 (16.7)	78.2 (15.0)	0.76
Symptoms	83.9 (17.3)	83.7 (14.4)	0.92
Daily activities	75.0 (21.9)	72.2 (20.4)	0.38
Treatment satisfactions	71.6 (18.6)	71.4 (17.8)	0.94
Treatment concerns	80.7 (17.5)	81.7 (16.9)	0.68
**1-year**			
Overall summary	81.9 (15.0)	82.8 (17.2)	0.72
Symptoms	87.5 (13.6)	90.8 (12.1)	0.13
Daily activities	77.1 (20.7)	76.0 (23.9)	0.76
Treatment satisfactions	74.1 (17.0)	76.4 (18.6)	0.44
Treatment concerns	84.3 (14.7)	86.2 (16.2)	0.42

Values are mean (SD), median (IQR), or n (%). ACE-I, angiotensin converting enzyme inhibitor; AF, atrial fibrillation; AFEQT, Atrial Fibrillation Effect on Quality-of-Life; ARB, angiotensin receptor blocker; BNP, brain natriuretic peptide; DOAC, direct oral anticoagulant; eGFR, estimated glomerular filtration rate; GI, gastrointestinal; LVDD, left ventricular end-diastolic dimension; LVEF, left ventricular ejection fraction; mHAS-BLED; modified HAS-BLED.

Table S4. Crude incidence of adverse clinical events during 2 years after registration

	OAC continuation	OAC discontinuation
All-cause death	26 (2.0%)	11 (2.8%)
Stroke/TIA	13 (1.0%)	4 (1.0%)
Bleeding	39 (3.1%)	10 (2.5%)

OAC, oral anticoagulant; TIA transient ischemic attack.

Table S5. Association OAC discontinuation and clinical adverse events during 2 years after registration

Outcomes	Hazard ratio (95% confidence interval)	P-value
All-cause death	1.03 (0.29-3.67)	0.96
Stroke/TIA	0.95 (0.26-3.39)	0.93
Bleeding	0.90 (0.44-1.84)	0.78

OAC, oral anticoagulant; TIA transient ischemic attack.
